# Transition Failure in Pediatric Inflammatory Bowel Disease: An Underrecognized Determinant of Long-Term Outcomes

**DOI:** 10.3390/children13091270

**Published:** 2026-09-18

**Authors:** Frank Risto Rommel, Stefan Schumann, Stefanie Weber, Andreas Jenke

**Affiliations:** 1 Department of General Pediatrics, University Children’s Hospital Marburg, University of Marburg, 35408 Marburg, Germany; 2 Department of General Pediatrics and Neonatology, University of Giessen, 35392 Giessen, Germany; 3 DRK-Kinderklinik Siegen, 57072 Siegen, Germany; 4 Department of Neonatology and Pediatric Gastroenterology, Children’s Hospital Kassel, Klinikum Kassel, 34125 Kassel, Germany; 5 Department of Medicine, Faculty of Health, University of Witten/Herdecke, 58455 Witten, Germany

**Keywords:** inflammatory bowel disease, transition to adult care, transition failure, paediatric gastroenterology, adherence, outcome framework, adolescent health, continuity of care

## Abstract

**Highlights:**

**What are the main findings?**

• Transition failure in paediatric IBD is conceptualised as a provisional composite outcome comprising five evidence-informed domains—non-adherence, disease exacerbation, emergency department utilisation, loss to follow-up, and psychosocial deterioration. For research purposes, fulfilment of ≥2 domains within 24 months post-transfer is proposed as a candidate classification rule rather than a validated diagnostic threshold.
• Existing outcome studies consistently demonstrate that unstructured transition is associated with significantly higher rates of disease flares, emergency admissions, and non-adherence compared with structured programmes, but no single study has simultaneously captured all five proposed domains, underscoring the need for prospectively designed validation studies.

**What are the implications of the main findings?**

• The transition failure framework provides a hypothesis-generating research and quality-assessment construct that could, after prospective validation, support benchmarking across institutions and healthcare systems, risk stratification before transfer, and standardised outcome evaluation.
• Effective transition requires structured, risk-stratified programmes that embed psychosocial assessment and graduated parental withdrawal as standard components, not optional add-ons; future outcome research must explicitly adjust for baseline disease activity and treatment refractoriness at transfer to avoid attributing disease-driven events to transition quality.

**Abstract:**

**Background/Objectives**: The transition from paediatric to adult care in inflammatory bowel disease (IBD) is a period of genuine clinical vulnerability. Despite international guidelines, real-world implementation of transition programmes remains inconsistent and the field lacks consensus on clinically meaningful outcome measures. We introduce the concept of “transition failure” as a provisional composite outcome framework to reorient research toward patient-centred endpoints. **Methods**: A narrative search of PubMed, MEDLINE, and Embase was conducted using terms including “inflammatory bowel disease”, “transition”, “transfer to adult care”, and “adherence”; no formal quality appraisal was applied. The search covered the databases from inception to 30 June 2026. **Results**: We synthesise current evidence on transition readiness and its limitations, delineate patient- and system-level barriers—including the underappreciated role of parental involvement—and characterise high-risk subgroups. Five evidence-informed, provisional domains of transition failure are proposed. Where published data permit, illustrative quantitative benchmarks are described; however, these are not assumed to be universally applicable across therapies or disease phenotypes. A composite of ≥2 domains within 24 months is proposed as a candidate research classification rule pending prospective validation, rather than as a clinical diagnostic threshold. **Conclusions**: The transition failure framework provides a hypothesis-generating construct for evaluating transition quality. Prospective multicentre validation, standardised and therapy-specific outcome definitions, adjustment for baseline disease severity, and risk-stratified multidisciplinary programmes are identified as research priorities.

## 1. Introduction

Inflammatory bowel disease (IBD), comprising Crohn’s disease and ulcerative colitis, is diagnosed during childhood or adolescence in a growing proportion of affected individuals worldwide. A systematic review of global epidemiological trends demonstrated a rising incidence of pediatric-onset IBD across the majority of reporting countries over the first two decades of this century [1]. Compared with adult-onset disease, pediatric IBD is characterized by more extensive bowel distribution at diagnosis, higher inflammatory burden, and earlier requirement for immunomodulatory or biologic therapy—features reflected in the ECCO-ESPGHAN guidelines on the medical management of paediatric Crohn’s disease [2]. Given its chronic, relapsing course, IBD necessitates lifelong medical management, making continuity of care a fundamental determinant of long-term disease trajectory.

The transition from paediatric to adult healthcare represents a critical juncture in this trajectory. Adolescents must progressively assume responsibility for disease self-management while navigating competing demands—educational transitions, identity development, and growing autonomy from parental oversight. These individual-level challenges interact with structural characteristics of healthcare systems frequently ill-equipped to support uninterrupted care continuity across the paediatric-adult divide.

**Figure 1:**
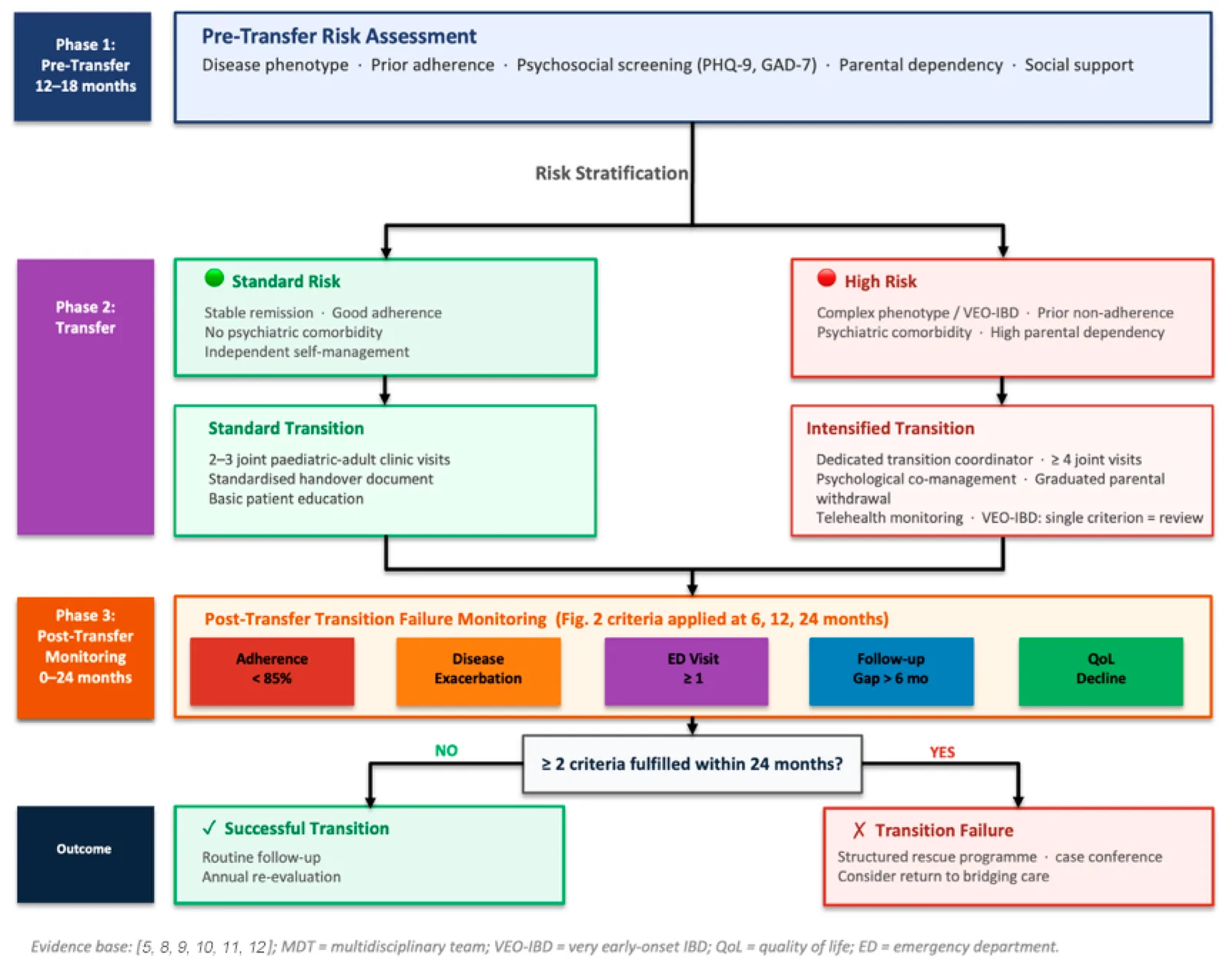
Clinical transition pathway with integrated transition failure monitoring [5,8,9,10,11,12]. The pathway begins with a structured pre-transfer risk assessment 12–18 months before transfer, evaluating disease phenotype, prior adherence, psychosocial status, parental dependency, and social support. Patients are stratified into standard- and high-risk groups, directing them toward standard or intensified transition programmes respectively. Following transfer, all patients undergo systematic post-transfer monitoring at 6, 12, and 24 months using the five evidence-informed transition failure criteria (Figure 2). Fulfilment of ≥2 criteria within 24 months defines transition failure and triggers a structured rescue intervention. For patients with very-early-onset IBD (VEO-IBD), any single criterion warrants immediate clinical review. ED = emergency department; MDT = multidisciplinary team; QoL = quality of life; VEO-IBD = very early-onset inflammatory bowel disease.

Guidelines addressing transitional care in IBD have been developed at European, British, and Australasian level [3,4,5]. Crowley et al., reviewing transitional care programmes across multiple chronic diseases, found that poorly managed transitions are consistently associated with adverse health consequences—a finding directly applicable to IBD [6]. Despite sustained international attention, implementation of structured transition programmes remains heterogeneous and the optimal programme format remains undefined [7]. The published literature has predominantly focused on transition readiness as a process measure rather than on clinical endpoints such as disease activity or hospitalisation rates, limiting the ability to assess the true impact of transition quality. Against this background, we propose the concept of transition failure as a unifying clinical framework to reorient research and practice toward measurable, patient-centred endpoints, operationalised in the clinical pathway presented in Figure 1.

**Figure 2:**
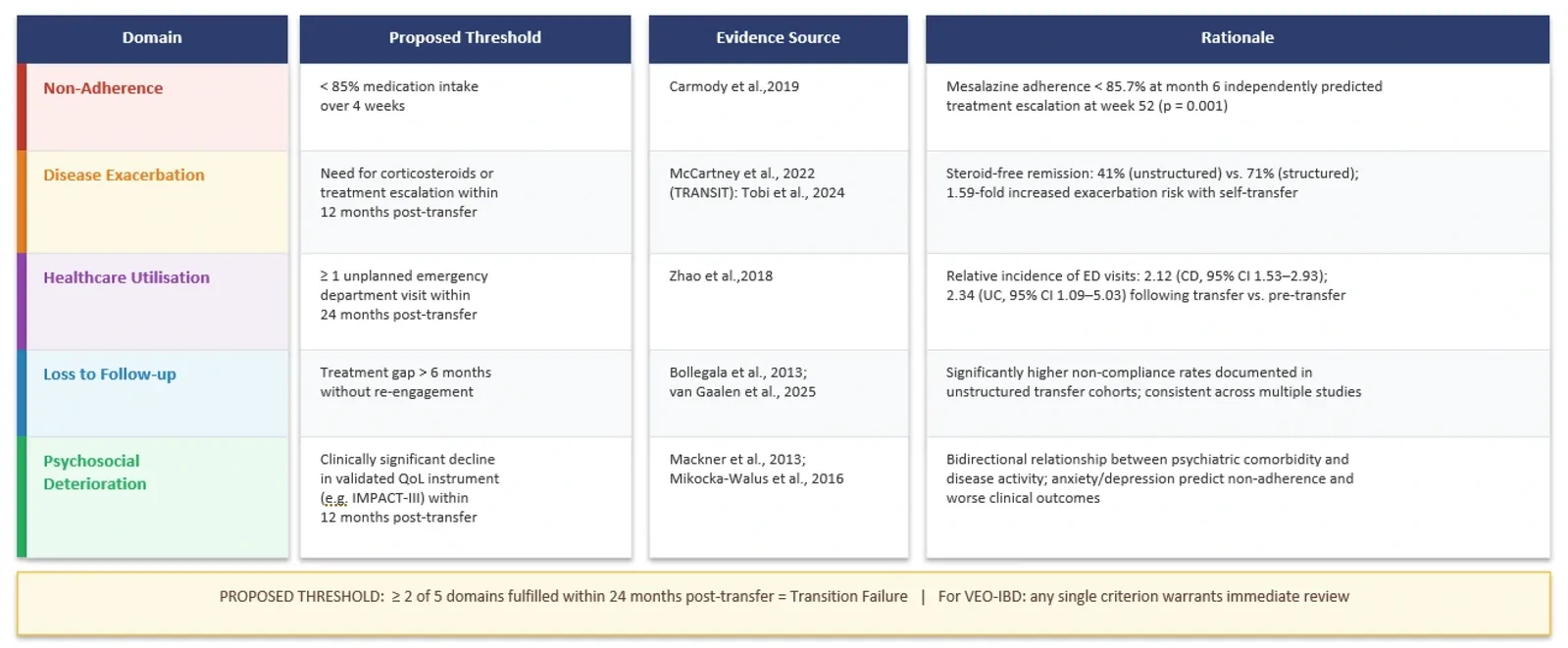
Evidence-informed, provisional criteria for transition failure in pediatric IBD [8,9,10,11,12,13,14,15]. Five clinical domains constitute the proposed transition failure framework, each anchored to an evidence-informed, provisional quantitative threshold derived from published outcome data. A composite of ≥2 domains fulfilled within 24 months post-transfer is proposed as the diagnostic threshold for transition failure, pending prospective validation. For patients with very-early-onset IBD (VEO-IBD), fulfilment of any single criterion warrants immediate clinical review. CD = Crohn’s disease; ED = emergency department; QoL = quality of life; UC = ulcerative colitis; VEO-IBD = very early-onset inflammatory bowel disease.

## 2. Materials and Methods/Literature Search Strategy

A narrative search of PubMed, MEDLINE, and Embase was conducted in June 2026 using the terms “inflammatory bowel disease”, “IBD”, “transition”, “transfer to adult care”, “adherence”, and “transition readiness”, combined using Boolean operators. The search covered each database from inception to 30 June 2026. Reference lists of included articles were manually screened for additional sources. No formal quality appraisal or risk-of-bias assessment was applied, consistent with the narrative and hypothesis-generating purpose of this review. The selection of studies reflects the authors’ interpretive judgements and may not represent all available evidence on each topic.

## 3. Results

### 3.1. Clinical Consequence and Definition of Transition Failure

It is reasonable and suggested by several studies that inadequate transition is associated with adverse outcomes, including increased post-transfer emergency department use, non-compliance, and more active disease [8,13]. In consequence, structured transition appears protective, with fewer flares, higher steroid-free remission, fewer emergency admissions, and reduced exacerbation risk compared with self-transfer [9,10].

A fundamental limitation, however, of current transition research is the absence of standardised, clinically grounded outcome definitions. Most studies rely on surrogate endpoints—transition readiness scores, patient satisfaction, or disease knowledge assessments—that have not been demonstrated to predict post-transfer clinical outcomes reliably [16,17]. A scoping review by Bihari et al. found that 26 eligible studies used heterogeneous outcome domains, frequently conflating process measures with clinical endpoints, and confirmed that no consensus definition of successful or unsuccessful transition currently exists in the IBD literature [18].

We propose transition failure as a multidimensional, hypothesis-generating outcome construct comprising five domains: loss to follow-up within 24 months of transfer; clinically meaningful non-adherence to prescribed therapy; disease exacerbation or need for treatment escalation within 12–24 months post-transfer; meaningful deterioration in psychosocial well-being or health-related quality of life; and ≥1 unplanned emergency department presentation within 24 months. The term “failure” refers to failure of the transition process to maintain safe continuity of care and is not intended to imply culpability of the adolescent or family.

The five domains are supported to different degrees by the published IBD transition literature, but the available evidence does not justify universal quantitative thresholds for every domain. Carmody et al. demonstrated in a prospective paediatric ulcerative colitis cohort that mesalazine adherence ≤85.7% at month six was associated with subsequent treatment escalation (*p* = 0.001) [11]. We therefore regard 85.7% as an illustrative benchmark for electronically monitored oral mesalazine adherence only, not as a general cut-off for IBD therapy. In future validation studies, non-adherence should be operationalised according to treatment modality: for oral therapies using validated self-report, pharmacy refill or electronic-monitoring measures; for self-injected biologics using missed or delayed doses and refill gaps; and for infusion therapies using missed or substantially delayed scheduled administrations. Drug concentrations may support assessment in selected therapies but should not be treated as a direct adherence measure because pharmacokinetic variability and immunogenicity also influence levels. For disease exacerbation, the TRANSIT study reported steroid-free remission rates of 41% versus 71% and emergency admission rates of 18% versus 5% in unstructured versus structured transition groups [10]. Zhao et al. documented increased post-transfer emergency department utilisation [8], while Bollegala et al. reported more frequent treatment gaps during transfer [13]. For psychosocial deterioration, validated quality-of-life or mental-health instruments provide the most reproducible approach [14,15]. These data support the domains conceptually, but prospective work is required to define therapy- and context-specific thresholds.

For hypothesis generation, we propose that fulfilment of ≥2 of the five domains within 24 months may classify a patient as having experienced transition failure. This choice is pragmatic rather than mathematically derived: requiring more than one domain is intended to reduce false-positive classification from isolated events that may occur for reasons unrelated to transition, while retaining sensitivity to clinically important clusters of adverse outcomes. We cannot infer from the current literature that ≥2 is superior to alternative rules such as ≥1, ≥3, differential weighting, or a continuous score. Accordingly, the proposed cut-off should be tested rather than assumed. Prospective validation should compare alternative thresholds using prespecified external outcomes and discrimination/calibration analyses, and should examine whether weighting domains or modelling them as correlated events improves performance. The 24-month window is similarly provisional and reflects the concentration of adverse events reported in the early post-transfer period [9,10].

Several important caveats apply to the proposed framework. The domains and candidate classification rule were not derived through a formal consensus process, Delphi study, ROC analysis, or prospective statistical modelling; equal weighting is therefore a pragmatic and untested assumption. The domains are also not statistically or clinically independent. Non-adherence may precipitate disease exacerbation, which may lead to emergency utilisation, psychosocial deterioration, and subsequent disengagement from care. Counting these events separately may therefore amplify a single causal cascade. Future validation should quantify inter-domain correlations and temporal sequencing and compare a simple count score with alternative approaches such as hierarchical outcomes, weighted scores, latent-variable models, or analyses that distinguish upstream process failures from downstream clinical consequences. Baseline disease activity is a further critical confounder: a flare or treatment escalation after transfer may represent refractory IBD rather than deficient transition care. Prospective studies should therefore record disease activity at transfer, inflammatory biomarkers, corticosteroid exposure, prior surgery, current and prior advanced therapies, recent exacerbations, and planned treatment escalation, and adjust for these variables in multivariable models. Sensitivity analyses should separately evaluate patients transferred in remission and those with active or refractory disease. Where feasible, independent adjudication should classify post-transfer events as primarily disease-driven, transition-related, system-related, or indeterminate. The emergency department criterion may also have limited individual-level specificity; a single unrelated emergency presentation could produce false-positive classification, particularly when combined with another weakly specific domain. These limitations reinforce that the framework is hypothesis-generating and should not be used for individual clinical diagnosis before prospective validation.

The application of the proposed transition-failure criteria to the existing literature, summarised in Table 1, highlights a central methodological limitation of the current evidence base: most available studies are not designed to determine whether transition failure, as a composite clinical outcome, has occurred. Several studies assess only a single domain, such as emergency healthcare utilisation in Zhao et al. or medication adherence in Carmody et al., while others provide indirect or domain-specific evidence rather than comprehensive post-transfer outcome assessment [8,11]. Even studies directly addressing transition often omit key domains such as psychosocial deterioration, objectively defined loss to follow-up, or medication adherence measured against clinically validated thresholds. As a result, most published studies can support individual components of the transition-failure framework but cannot independently confirm or exclude transition failure according to the proposed ≥2-domain definition. The clearest evidence emerges from studies comparing structured transition with self-transfer or unstructured transfer, particularly Tóbi et al. and McCartney et al., in which multiple domains are assessed, and the comparator groups fulfil criteria consistent with transition failure [9,10]. It is therefore clear that the field requires prospective studies specifically designed to capture all five domains within a standardised post-transfer follow-up window, rather than relying on heterogeneous single-outcome studies that are insufficient to answer the broader question of transition failure.

If prospectively validated, the practical utility of this framework may lie in complementing, rather than replacing, the Transition Success Score (TSS) developed by van Gaalen et al. [12]. The TSS was derived from an international Delphi process and prospectively validated as a quantitative measure of transition success, with substantial emphasis on self-management, healthcare navigation, appointment attendance, medication adherence, satisfaction, and quality of life. Our proposed framework overlaps with the TSS in adherence, continuity of care, and psychosocial outcome, but differs by deliberately foregrounding adverse clinical and healthcare-utilisation events such as exacerbations and emergency presentations. Importantly, the transition failure construct should not simply be interpreted as the mathematical inverse of the TSS: a patient may demonstrate good self-management skills yet experience disease-driven clinical deterioration, whereas another may have poor engagement without an early flare. Future prospective studies should therefore administer the TSS alongside the proposed failure domains to test convergent and discriminant validity and determine whether the adverse-outcome framework adds information beyond the lower end of the validated TSS. Only if incremental validity is demonstrated would a separate transition-failure metric be justified.

### 3.2. Transition Readiness: Current Evidence and Limitations

One additional problem is the concept of transition readiness. It has been a research construct for over a decade, typically operationalized through structured instruments assessing disease knowledge, self-management skills, and healthcare navigation competencies. A comprehensive review by Fishman and Brannigan concluded that while readiness tools are increasingly incorporated into clinical practice, their predictive validity for post-transfer clinical outcomes remains inadequately established [19].

Fishman et al. provided an important empirical calibration: evaluating adult IBD patients as a reference standard, the study found substantial medication knowledge gaps even among established patients—suggesting that benchmarks applied in adolescent readiness assessment may be systematically too low [17]. Fishman et al. subsequently demonstrated that informal provider education on transition had no measurable effect on patient self-management skills [20]. Gray et al. found that even older adolescents routinely approach transfer without demonstrating independent management of key healthcare tasks [21]. Philpott and Kurowski identified a persistent gap between readiness assessment and clinical outcome data [22].

A further limitation is the well-documented discrepancy between perceived and objective competence: adolescents frequently report high confidence while demonstrating substantive gaps in medication knowledge, monitoring requirements, and alarm symptom recognition [17]. Static readiness measures also fail to capture dynamic psychosocial factors that profoundly influence adherence and care engagement in real-world settings. Viewed through the lens of the transition failure framework, this limitation becomes particularly clear: none of the five evidence-informed criteria in Figure 2 are directly captured by standard readiness instruments. Huang et al. demonstrated that self-reported transition readiness checklists showed poor concordance with objective skills-based assessment in an IBD cohort, with adolescents systematically overestimating their own competencies [23], further undermining self-report readiness as a standalone proxy for post-transfer outcomes. A young person may score adequately on a readiness questionnaire while already fulfilling one or more transition failure criteria—a scenario invisible to clinicians relying on readiness scores alone.

### 3.3. System-Level Barriers

Patient-level vulnerabilities interact with structural features of healthcare systems that frequently impede effective transition. The systematic review by Erős et al., encompassing 23 studies, documented substantial variability in programme components, organisational models, and outcome measures across institutions [24]. International surveys and guideline development processes have highlighted wide heterogeneity in transfer timing, communication protocols, and availability of dedicated transition resources [3,4]. The Australasian consensus guidelines identified structured transition programmes, designated transition coordinators, standardised mental health assessment, and individualised clinical handover as priority recommendations—underscoring that these basic structural components are not yet universally in place [5].

This heterogeneity makes transition failure difficult to analyse across studies, particularly because healthcare systems differ substantially between countries in referral pathways, insurance structures, availability of specialist adult IBD services, and integration between paediatric and adult care. The same outcome may therefore have different meanings across settings: an emergency presentation, delayed appointment, or medication interruption may reflect patient disengagement in one system but structural access barriers in another. Future studies should therefore report healthcare-system context alongside clinical outcomes, allowing transition failure to be distinguished from failures of service organisation.

### 3.4. High-Risk Subgroups

Recognition that transition failure disproportionately affects specific patient subgroups is essential for rational intervention design, as reflected in the risk-stratified pathway of Figure 1. Patients with complex disease phenotypes—those requiring biologic therapy, those with prior surgical history, or frequent relapses—require more intensive monitoring and face greater consequences from care discontinuity.

Very-early-onset IBD (VEO-IBD), defined by diagnosis before six years of age, constitutes a biologically and clinically distinct entity warranting individualised transition planning. As reviewed by Kelsen et al., VEO-IBD is characterised by marked genetic heterogeneity, frequent monogenic aetiology, and severe disease courses often refractory to conventional therapies [25]. Uhlig et al. and Kammermeier et al. emphasised the complexity of precision medicine approaches and the critical importance of sustained multidisciplinary genomic care pathways across the transition [26,27]. For VEO-IBD patients, the standard transition failure threshold of ≥2 criteria cannot be applied in the same way; given the severity of their underlying condition, even a single criterion fulfilled within 24 months should be considered an indication for immediate review.

Psychosocial vulnerability represents a further underestimated risk dimension. The NASPGHAN clinical report identified elevated rates of depressive and anxiety disorders, impaired quality of life, and compromised social functioning as prevalent features of the paediatric IBD population [14]. The bidirectional relationship between psychiatric comorbidity and disease activity means that adolescents entering adult care with untreated mental health problems face a consequential cascade: disengagement drives non-adherence, which drives disease flare, which further erodes care engagement [15,28]. This pattern represents the sequential fulfilment of multiple transition failure domains within a short timeframe—precisely the scenario the monitoring framework in Figure 1 is designed to detect and interrupt.

An underappreciated dimension of transition risk concerns parental involvement and its gradual, often poorly managed withdrawal. In paediatric IBD care, parents typically manage medication procurement, attend clinic visits, and communicate with the healthcare team. Mackner et al. noted that family functioning is closely linked to treatment adherence [14]. Fishman et al. demonstrated that even older adolescents defer core healthcare tasks to their parents [17]. Bihari et al. found that parents experienced their own transition—one requiring relinquishment of oversight roles occupied for years, frequently without guidance from healthcare teams [29]. Programmes that explicitly address the parental transition through graduated withdrawal of management tasks, monitored against concrete milestones, may prove as clinically important as those targeting patient readiness directly.

### 3.5. Structured Transition Programmes: Current Approaches and Evidence

The evidence base for transition interventions has matured over the last decade. Structured programmes incorporating joint paediatric-adult visits, dedicated coordinators, and systematic readiness assessment now have empirical outcome support. The TRANSIT study and the Tóbi longitudinal cohort (2024) provide the strongest evidence to date that structured transition reduces clinically meaningful harm [9,10]. Goodhand et al. earlier reported higher remission rates and better developmental outcomes in patients undergoing structured versus unstructured transfer [30].

The systematic review by Erős et al. found that outcomes improved in 11 of 23 evaluated studies, with joint visits and patient education emerging as the most employed components [24]. Chan et al., in the most recent systematic review of 29 studies, confirmed that transition-related and disease-related outcomes broadly benefit from structured programmes—but concluded that the optimal format remains undefined and methodological limitations remain significant [7].

The risk-stratified pathway illustrated in Figure 1 integrates these evidence components: pre-transfer risk assessment directs patients toward standard or intensified transition pathways, with post-transfer monitoring applying the transition failure criteria from Figure 2 at 6, 12, and 24 months. A composite score of ≥2 criteria triggers a structured rescue intervention. Digital health tools—including the myIBDcoach telemedicine platform evaluated by de Jong et al., which demonstrated significantly fewer hospital admissions compared with standard care in a randomised trial—support systematic, low-burden monitoring in the post-transfer phase, particularly for patients who have transferred to non-tertiary services [31].

## 4. Discussion

The central problem in IBD transition research is not a shortage of clinical attention or guideline development—it is the absence of an agreed definition of what an unsuccessful transition looks like. Without such a definition, studies have defaulted to measuring what is measurable rather than what is meaningful: transition readiness scores, programme satisfaction, and process completion rates. Transition readiness has not proven reliably predictive of post-transfer disease course or healthcare utilization [16,17,19]. At the same time, the validated Transition Success Score (TSS) now provides an important consensus-derived measure of the positive end of transition outcome [12]. Our proposal should therefore be viewed as a complementary hypothesis: whether a specifically adverse-outcome construct adds clinically relevant information beyond the lower range of the TSS remains unknown and requires direct comparative validation. The emergence of outcome-oriented evidence from the TRANSIT study, the Hungarian longitudinal cohort, and the Dutch TSS validation provides part of the empirical basis for such work. The BUTTERFLY multicentre study (n = 278, 34 Spanish centres) corroborated that structured transition reduced post-transfer flare rates (22% vs. 36%) and hospitalisation (3% vs. 10%), but also demonstrated that active IBD, low BMI, and corticosteroid use at transfer were independent predictors of poor outcome regardless of transition structure [32]. Bennett et al. showed that structured transition improved service engagement but found no significant differences in biologic failure, steroid requirement, or surgical intervention between cohorts [33]. Together, these findings underscore that post-transfer clinical events cannot be attributed to transition quality without accounting for baseline disease burden.

The transition failure framework represents the next logical step: synthesising existing outcome data into a standardised composite definition that can serve as a common currency for future research and a practical quality indicator for clinical services. A centre-level transition failure rate would enable direct comparison across programmes and healthcare contexts, identification of effective programme components, and prospective targeting of high-risk patients. This cross-institutional comparison is not currently possible given the heterogeneous outcomes in the existing literature as documented by Bihari et al. [18].

The role of mental health deserves particular emphasis. The bidirectional relationship between psychiatric comorbidity and disease activity means that adolescents with untreated mental health problems face a cascade whose sequential progression—disengagement, non-adherence, disease flare, further disengagement—represents the simultaneous or rapid sequential fulfilment of multiple transition failure domains [15,28]. This is the pattern the monitoring pathway in Figure 1 is designed to interrupt before it completes. Psychological assessment and support must be embedded as structural components of transition care, as explicitly recommended by Vernon-Roberts et al. [5].

The quantitative burden of psychosocial impairment in paediatric IBD lends further weight to this recommendation. A meta-analysis by Greenley et al. confirmed that psychosocial functioning is significantly impaired across multiple domains in youth with IBD compared with healthy controls, with effect sizes of clinical relevance across internalising symptoms, social competence, and quality of life [34]. Szigethy et al. further characterised depression subtypes in this population and demonstrated that distinct subtypes show differential associations with inflammatory markers and disease severity—indicating that psychiatric comorbidity in paediatric IBD is not a uniform reactive phenomenon but a multidimensional condition requiring structured, phenotype-sensitive assessment [35]. For transition programmes, this implies that a single screening score is insufficient: depression subtype, inflammatory status, and social functioning must be evaluated in combination to identify patients at highest psychosocial risk at the point of transfer.

Comparative perspectives from adjacent fields provide instructive reference points. Crowley et al. found that the most consistently successful transition programmes across chronic conditions involved dedicated clinics jointly staffed by paediatric and adult physicians—a finding corroborated in IBD by the TRANSIT study, with further support from retrospective IBD-specific studies demonstrating improved disease control, medication adherence, and care retention following structured versus unstructured transfer [6,36]. In type 1 diabetes, Spaic et al. demonstrated that structured transition improved clinic attendance and reduced distress, while Lotstein et al. documented a 2.5-fold increased risk of poor glycaemic control following transfer—a quantitative parallel to the emergency utilisation data in IBD [8,37,38]. The IBD field is now in a position to build on these precedents by prospectively validating the transition failure criteria proposed here.

Risk stratification offers a pragmatic pathway toward more efficient resource allocation, but pre-transfer risk factors should be distinguished from post-transfer outcome domains. The proposed failure domains are intended primarily as post-transfer outcomes; they should not automatically be treated as validated pre-transfer screening thresholds. Instead, prospective cohorts should evaluate candidate baseline predictors including prior non-adherence, active disease, recent corticosteroid exposure, previous surgery, repeated biologic failure, psychosocial vulnerability, parental dependency, and prior missed appointments. Patient-level barriers to medication adherence in adolescents with IBD—including forgetfulness, concerns about side effects, and inadequate social support structures—are well characterised and represent modifiable targets for pre-transfer intervention [39]. A future risk model should be developed and validated separately from the outcome definition, ideally in international multicentre cohorts with prespecified predictors and external validation.

## 5. Conclusions

The transition from paediatric to adult care in IBD is a period of clinically relevant vulnerability. An emerging body of evidence suggests that structured transition with joint clinical involvement can improve care continuity and may reduce flares and emergency utilisation. The concept of transition failure, as operationalised in this review, is proposed as a hypothesis-generating adverse-outcome framework rather than a validated diagnostic instrument. It is intended to complement existing success-oriented measures such as the TSS by focusing on clinically consequential events that may occur after transfer.

The proposed domains and the candidate ≥2-domain classification rule require prospective validation before any clinical or benchmarking use. Future international multicentre cohorts should collect all domains using standardised definitions, administer the validated TSS in parallel, and record baseline disease activity, biomarkers, corticosteroid exposure, treatment history, surgery, psychosocial status, and healthcare-system context. Analyses should test alternative thresholds and weighting schemes, quantify correlations and temporal relationships among domains, adjust for baseline severity using multivariable and centre-level models, and perform sensitivity analyses in patients transferred in remission versus active disease. Such studies should also assess false-positive classification at the individual-patient level and determine whether the proposed framework adds predictive or evaluative information beyond the TSS. The term transition failure is used here to describe an adverse outcome of the transition process or care system and should never be interpreted as assigning blame to the adolescent or family.

## 6. Limitations

Several methodological considerations should be acknowledged. The literature search was not pre-registered and no formal quality appraisal or risk-of-bias assessment was applied; publication bias towards positive transition outcomes cannot be excluded. The transition failure framework is explicitly provisional and hypothesis-generating. Neither the individual domain thresholds nor the candidate ≥ 2 of 5 rule were derived by Delphi consensus, ROC analysis, prospective statistical modelling, or external validation, and equal weighting is an untested pragmatic assumption. The domains may be causally and statistically correlated, so a simple count can double-count different stages of the same adverse cascade. The mesalazine adherence value of ≤85.7% is therapy-specific and should not be generalised to biologics, immunomodulators, or Crohn’s disease; future studies require modality-specific adherence definitions. Baseline disease severity is a critical confounder: without adjustment, a post-transfer flare or treatment escalation cannot reliably distinguish transition-related harm from refractory disease. Prospective validation must therefore prespecify disease-activity and treatment-history covariates, use multivariable and preferably multilevel models, and include sensitivity analyses stratified by remission versus active disease at transfer. The emergency department domain may have limited individual-level specificity and may contribute to false-positive classification when presentations are unrelated to IBD or transition. Finally, healthcare systems differ in referral pathways, insurance structures, specialist availability, and paediatric-adult interface models; an emergency presentation, care gap, or medication interruption may reflect patient disengagement in one setting and structural access failure in another. These limitations preclude use of the framework as a clinical diagnostic instrument at present.

## Figures and Tables

**Table 1:** Application of Transition-Failure Criteria Across Selected Pediatric-Onset IBD Studies. For each study, the five domains of the transition failure framework are assessed based on reported outcome data and classified as assessed, partly assessed, or not assessed. Studies are included if they reported at least one domain-relevant outcome in a paediatric IBD transition context. The table illustrates that no existing study captures all five domains within a standardised post-transfer follow-up window, and that the composite transition failure threshold of ≥2 domains can therefore not be applied retrospectively to most available evidence. ED = emergency department; IBD = inflammatory bowel disease; QoL = quality of life.

Transition-Failure Domain	Tóbi et al., 2024 [9]	Zhao et al., 2018 [8]	Carmody et al., 2019 [11]	McCartney et al., 2022 [10]	Bollegala et al., 2013 [13]	Mikocka-Walus et al., 2016 [15]
**Non-adherence**	**Assessed.** Worse adherence in self-transfer; non-adherence 31.9% vs. 16.4% with structured transition.	**Not assessed.** No medication-intake or adherence outcome.	**Assessed.** Mesalazine adherence < 85.7% predicted treatment escalation at week 52.	**Not clearly assessed.** Medication adherence not a main reported outcome.	**Assessed.** Documented noncompliance increased after transfer/adult care.	**Not assessed.** Review focused on anxiety and depression, not adherence.
**Disease exacerbation**	**Assessed.** Self-transfer had 1.88-fold higher relapse risk; structured transition had more time in remission.	**Partly assessed.** Hospitalisations did not significantly increase; relapse, steroid use, and escalation not measured.	**Assessed.** Non-adherence predicted later treatment escalation in paediatric UC.	**Assessed.** Steroid-free outcome better with structured transition: 71% vs. 41%.	**Assessed.** Disease activity appeared lower in adult care; no clear exacerbation signal.	**Partly assessed.** Anxiety/depression associated with active IBD, but not post-transfer relapse.
**Healthcare utilisation**	**Partly assessed.** Healthcare engagement assessed, but ≥1 unplanned ED visit within 24 months not clearly reported.	**Assessed.** ED visits increased after transfer: CD RI 2.12; UC RI 2.34.	**Not assessed.** No ED or transition-related utilisation outcome.	**Assessed.** ED visits leading to admission lower with structured transition: 5% vs. 18%.	**Partly assessed.** Resource use changed, but unplanned ED visits within 24 months not clearly reported.	**Not assessed.** No ED, hospitalisation, or resource-use endpoint.
**Loss to follow-up**	**Assessed.** Self-transfer had 1.59-fold higher risk of discontinuing care.	**Not assessed.** No >6-month treatment-gap outcome.	**Not assessed.** No transition follow-up outcome.	**Not clearly assessed.** No defined >6-month treatment gap reported.	**Partly assessed.** Lower outpatient follow-up may suggest disengagement, but no defined >6-month gap.	**Not assessed.** No transition follow-up outcome.
**Psychosocial deterioration**	**Not assessed.** No QoL/anxiety/depression deterioration outcome.	**Not assessed.** Administrative data lacked psychosocial outcomes.	**Not assessed.** No QoL or psychosocial outcome.	**Not assessed.** No validated psychosocial deterioration endpoint in this outcome paper.	**Partly assessed.** Psychiatric comorbidity recorded, but no validated decline outcome.	**Assessed.** Supports high anxiety and depression burden in IBD, but not post-transfer deterioration.

## Data Availability

No new data were created or analysed in this study. Data sharing is not applicable to this article.

## Abbreviations

The following abbreviations are used in this manuscript:

BMI	body mass index
CD	Crohn’s disease
CI	confidence interval
ECCO	European Crohn’s and Colitis Organisation
ED	emergency department
ESPGHAN	European Society for Paediatric Gastroenterology, Hepatology and Nutrition
GAD-7	Generalised Anxiety Disorder Assessment-7
IBD	inflammatory bowel disease
MDT	multidisciplinary team
NASPGHAN	North American Society for Pediatric Gastroenterology, Hepatology, and Nutrition
PHQ-9	Patient Health Questionnaire-9
QoL	quality of life
UC	ulcerative colitis
VEO-IBD	very early-onset inflammatory bowel disease
